# Minimally invasive versus open pancreatoduodenectomy—systematic review and meta-analysis

**DOI:** 10.1007/s00423-017-1583-8

**Published:** 2017-05-09

**Authors:** Michał Pędziwiatr, Piotr Małczak, Magdalena Pisarska, Piotr Major, Michał Wysocki, Tomasz Stefura, Andrzej Budzyński

**Affiliations:** 1 2nd Department of General Surgery, Jagiellonian University Medical College, Kopernika 21, 31-501 Krakow, Poland; 2 Department of Endoscopic, Metabolic and Soft Tissue Tumors Surgery, Kopernika 21, 31-501 Kraków, Poland; 3 Centre for Research, Training and Innovation in Surgery (CERTAIN Surgery), Kopernika 21, 31-501 Kraków, Poland

**Keywords:** Laparoscopy, Pancreatoduodenectomy, Pancreatic cancer, Robotic surgery, Whipple procedure

## Abstract

**Purpose:**

The purpose of this systematic review was to compare minimally invasive pancreatoduodenectomy (MIPD) versus open pancreatoduodenectomy (OPD) by using meta-analytical techniques.

**Methodology:**

Medline, Embase, and Cochrane Library were searched for eligible studies. Data from included studies were extracted for the following outcomes: operative time, overall morbidity, pancreatic fistula, delayed gastric emptying, blood loss, postoperative hemorrhage, yield of harvested lymph nodes, R1 rate, length of hospital stay, and readmissions. Random and fix effect meta-analyses were undertaken.

**Results:**

Initial reference search yielded 747 articles. Thorough evaluation resulted in 12 papers, which were analyzed. The total number of patients was 2186 (705 in MIPD group and 1481 in OPD). Although there were no differences in overall morbidity between groups, we noticed reduced blood loss, delayed gastric emptying, and length of hospital stay in favor of MIPD. In contrary, meta-analysis of operative time revealed significant differences in favor of open procedures. Remaining parameters did not differ among groups.

**Conclusion:**

Our review suggests that although MIPD takes longer, it may be associated with reduced blood loss, shortened LOS, and comparable rate of perioperative complications. Due to heterogeneity of included studies and differences in baseline characteristics between analyzed groups, the analysis of short-term oncological outcomes does not allow drawing unequivocal conclusions.

**Electronic supplementary material:**

The online version of this article (doi:10.1007/s00423-017-1583-8) contains supplementary material, which is available to authorized users.

## Introduction

It has been documented that minimally invasive surgery may be successfully and safely applied to practically every intraabdominal surgical operation. Although the first total laparoscopic pancreatoduodenectomy was described more than 20 years ago by Gagner and Pomp, it still has not become the gold standard in the treatment of periampullary malignancies [1]. Globally, there is a trend in oncological surgery towards the reduction of surgical trauma (introduction of minimally invasive surgery, changes in the operative technique, organ sparing procedures, modification of perioperative care) [2, 3]. Although there is a lack of randomized trials in the field of minimally invasive pancreatic head resections, a growing number of case series and cohort studies have been published comparing the safety and efficacy of minimally invasive and open pancreatic head resection [4]. In many centers performing laparoscopic or robotic pancreatic head resections, these procedures are in fact not minimally invasive pancreatoduodenectomies (MIPD) but rather cases in which the dissection is being performed in a minimally invasive manner, while anastomoses are performed manually [5–9]. This could potentially create a bias in the analysis of outcomes.

Since it has been shown that laparoscopic approach is feasible in other types of procedures in oncological surgery (e.g., gastric, colorectal), we hypothesized it might also be achievable in pancreatic head resections [10, 11]. Therefore, in this review, we aimed to systematically review the available published literature and conduct a meta-analysis comparing minimally invasive (laparoscopic or robotic) and open pancreatoduodenectomy.

## Methods

### Study selection

A systematic review of the literature was performed using the Medline, Embase, and Cochrane databases to identify all eligible studies that compared MIPD versus open pancreatoduodenectomy (OPD). The used search terms included “laparoscopy,” “minimally invasive,” “laparoscopic assisted,” “robotic,” robotic-assisted,” “pancreatoduodenectomy,” “pancreaticoduodenectomy,” “Whipple,” “Traverso,” “pancreatic head resection,” and “duodenopancreatectomy”. These terms were combined using Boolean operators “AND” and “OR”. References of the acquired articles were also hand-searched. Most recent search was done on 5th February, 2017. Ovid search strategy is available in supplementary file 1.

### Data extraction

All references were reviewed and evaluated by two researchers. In case of doubt about inclusion, a third reviewer was consulted until consensus was reached. Only full length articles were eligible for extraction. When included, the following data were extracted: first author, year of publication, study design, number of operated subjects, 30-day readmission, conversion rate, perioperative, and short-term oncological outcomes.

### Inclusion criteria

Studies eligible for further analysis had to fulfill the following criteria: (1) comparison of characteristics and perioperative outcomes of mini-invasive techniques (laparoscopic or robotic surgery) to open approach in patients undergoing pancreatoduodenectomy, (2) objective evaluation of operative time and reports of pancreatic fistula, and (3) no language restrictions were used.

### Exclusion criteria

Studies were excluded when (1) lack of comparative data, (2) hand-assisted technique in the description of surgical methodology (especially anastomoses) or surgical method was unclear, (3) lack of primary outcomes or insufficient data to analyze, (4) studies regarding techniques other than pancreatoduodenectomy, and (5) extraction of data only on pancreatoduodenectomy not possible.

### Outcomes of interest

MIPD (excluding hand-assisted) and OPD were compared on the basis of perioperative outcomes (operative time, intraoperative blood loss), postoperative complications (overall morbidity, pancreatic fistula, delayed gastric emptying, postoperative hemorrhage), oncologic safety (lymph node harvest, R1 rate), length of hospital stay, and 30-day readmission rate.

### Statistical analysis

Analysis was performed using RevMan 5.3 (freeware from The Cochrane Collaboration). Statistical heterogeneity and inconsistency were measured using Cochran’s Q tests and I2, respectively. Qualitative outcomes from individual studies were analyzed to assess individual and pooled risk ratios (RR) with pertinent 95% confidence intervals (CI) favoring minimally invasive over open pancreatoduodenectomy and by means of the Mantel-Haenszel fixed-effects method in the presence of low or moderate statistical inconsistency (I2 ≤ 10%) and by means of a random-effects method (which better accommodates clinical and statistical variations) in the presence of high statistical inconsistency (I2 > 10%). When appropriate, mean and standard deviation were calculated from medians and interquartile ranges using a method proposed by Hozo et al. [12]. Weighted mean differences (WMD) with 95% CI are presented for quantitative variables using the inverse variance fixed-effects or random-effects method. Statistical significance was observed with two-tailed 0.05 level for hypothesis and with 0.10 for heterogeneity testing, while unadjusted *p* values were reported accordingly. Non-randomized studies were evaluated by the Newcastle–Ottawa Scale (NOS), which consists of three factors: patient selections, comparability of the study groups, and assessment of outcomes. A score of 0 to 9 was assigned to each study, and studies achieving a score of 6 or greater were considered high quality. This study was performed according to the Preferred Reporting Items for Systematic reviews (PRISMA) guidelines and MOOSE consensus statement [13, 14].

## Results

**Fig. 1:**
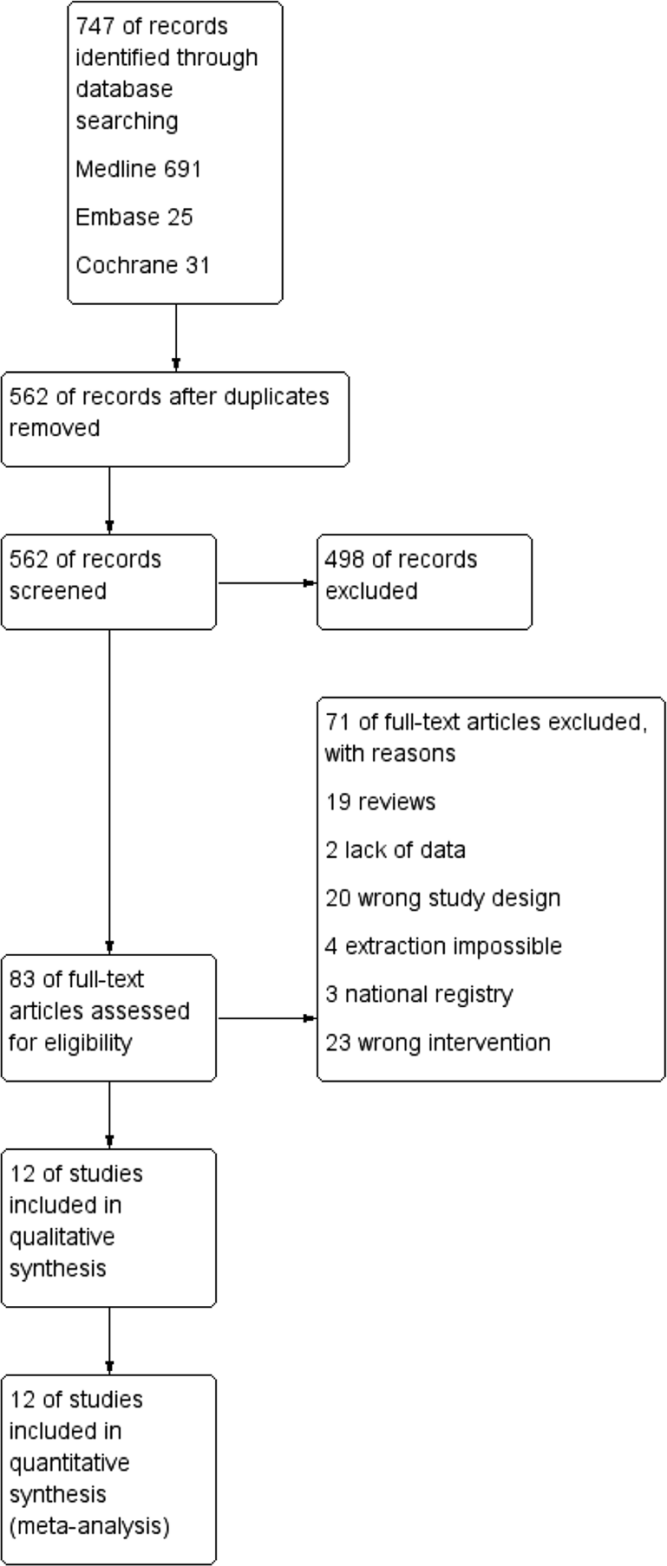
Flowchart of the studies

Initial reference search yielded 747 articles. After removing 185 duplicates, 562 articles had their titles and abstracts evaluated. This resulted in 83 papers suitable for full-text review, where 19 studies were review articles on the subject of pancreatoduodenectomy, 20 articles were excluded due to wrong study design, 23 because of the wrong type of intervention, three studies were based on national registries, and two did not provide sufficient data for inclusion. Four studies by Asbun et al., Stauffer et al. Mesleh et al., and Gumbs et al. met inclusion criteria; however, it was impossible to extract data on pancreatoduodenectomy alone. Besides, three of them included patients from the same pancreatic center treated in the overlapping period of time [15–18]. Thus, they were excluded from further analysis. Finally, we enrolled 12 studies with a total of 2186 patients (705 underwent MIPD and 1481 underwent open procedure) (Table 1) [19–30]. Quality of the analyzed studies according to NOS is high, with majority of studies scoring ≥7 out of 9. Authors were contacted to provide additional data on their studies. Flowchart of the analyzed studies is presented in Fig. 1.

**Table 1:** Study characteristics

Author	Year	Country	Study design	Type of intervention	Number of patients MIPD/OPD	Conversion rate	Neoadjuvant chemotherapy MIPD/OPD	Vein resection MIPD/OPD	Quality score (9 max.)
Baker et al. [19]	2016	USA	RS, SC	RPD	22/49	14%	11.1/12.5%	13.6/14.3%	7
Bao et al. [20]	2014	USA	RS, CM, SC	RPD	28/28	14%	ND	ND	6
Boggi et al. [21]	2016	Italy	RS, SC	RPD	83/36	2%	ND	8.4/11.1%	8
Buchs et al. [22]	2011	USA	RS, SC	RPD	44/39	ND	ND	ND	8
Chen et al. [23]	2015	China	PS, CM, SC	RPD	60/120	ND	ND	5.0/6.7%	8
Lai and Tang [24]	2012	China	RS, SC	RPD	20/67	5%	ND	ND	6
Zhou et al. [25]	2011	China	RS, SC	RPD	8/6	ND	ND	ND	8
Zureikat et al. [26]	2016	USA	RS, MC	RPD	211/817	4.7%	ND	ND	7
Croome et al. [27]	2014	USA	RS, SC	LPD	108/214	6%	11.1/14.0%	20.4/23.8%	8
Delitto et al. [28]	2016	USA	PS, SC	LPD	77/61	ND	5.8/6.0%	ND	7
Tan et al. [29]	2015	China	RS, MC	LPD	30/30	7%	ND	ND	6
Zureikat et al. [30]	2011	USA	RS, CM, SC	LPD	14/14	14%	ND	7.1/0.0%	7

*LPD* laparoscopic pancreatoduodenectomy, *RPD* robotic pancreatoduodenectomy, *OPD* open pancreatoduodenectomy, *RS* retrospective study, *PS* prospective study, *SC* single center, *MC* multi center, *CM* case matched, *ND* no data, *MIPD* minimally invasive pancreatoduodenectomy

The analysis revealed that MIPD group differed significantly in tumor size (reported in eight studies) in comparison to open approach (2.63 vs 3.26 cm, MD = −0.46 CI 95% −0.72–−0.20, *p =* 0.0005).

**Fig. 2:**
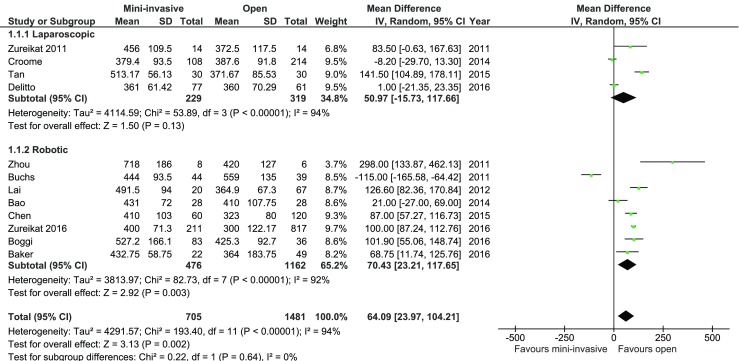
Pooled estimates of operative time comparing mini-invasive pancreatoduodenectomy versus open surgery. *CI* confidence interval, *df* degrees of freedom

The operative time was reported by all authors. The mean total operative time for MIPD was 464 min, whereas for open procedure, it was 388 min. A subgroup analysis showed a mean value of 427 min for laparoscopic pancreatoduodenectomy and 482 min for robotic procedures. The analysis (Fig. 2) showed that the operative time was significantly shorter in the open procedure group: MD = 64.09, 95% CI 23.97–104.21, *p* for effect = 0.002, *p* for heterogeneity <0.00001, *I* ^2^ = 94%. However, a subgroup analysis revealed no statistically significant differences in both laparoscopic and robotic subgroup, which, together with high heterogeneity, limits the quality of evidence of this outcome.

Conversions were reported by eight authors. There were 31 (6.01%) conversions in total, with 11 (7.23%) in laparoscopic group and 20 (5.49%) in robotic.

**Fig. 3:**
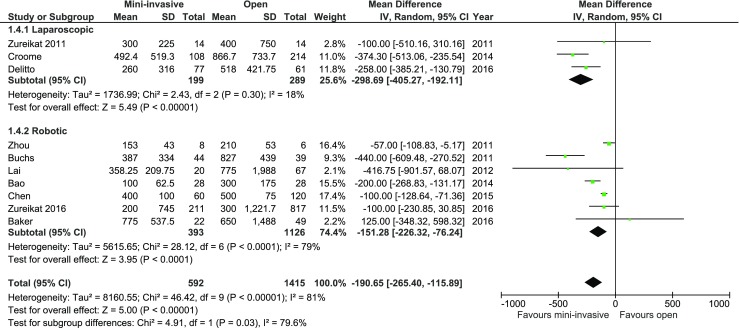
Pooled estimates of blood loss comparing mini-invasive pancreatoduodenectomy versus open surgery. *CI* confidence interval, *df* degrees of freedom

Data on blood loss was presented in 10 of 12 studies. All authors, except Baker et al., Lai et al., and Zureikat et al., reported significant differences in this outcome. Mean blood loss for laparoscopic group was 350.8, 339.04 ml for robotic group, and 342.57 ml for the whole MIPD group, whereas in the case of open procedure, it was 534.67 ml. The analysis (Fig. 3) revealed a significant difference in both subgroups, as well as in total: MD = −190.65, 95% CI −265.40–−115.89, *p* for effect <0.00001, *p* for heterogeneity <0.00001, *I* ^2^ = 81%.

**Fig. 4:**
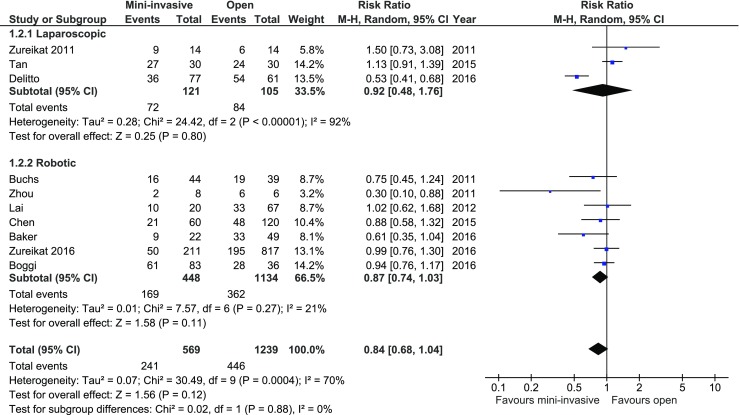
Pooled estimates of morbidity comparing mini-invasive pancreatoduodenectomy versus open surgery. *CI* confidence interval, *df* degrees of freedom

Overall morbidity was reported in 10 of 12 studies. Authors Bao and Croome were contacted to provide necessary data, with no response. Of all studies, only two authors, Delitto and Zhou, reported a significant difference in morbidity in analyzed groups. Meta-analysis of all included studies, as well as subgroup analysis, did not show statistically significant differences: 241/569 (42.36%) in MIPD and 446/1239 (36.00%) in the control group, *RR* = 0.84, 95% CI 0.68–1.04, *p* for effect = 0.12, *p* for heterogeneity = 0.0004, *I* ^2^ = 70% (Fig. 4).

**Fig. 5:**
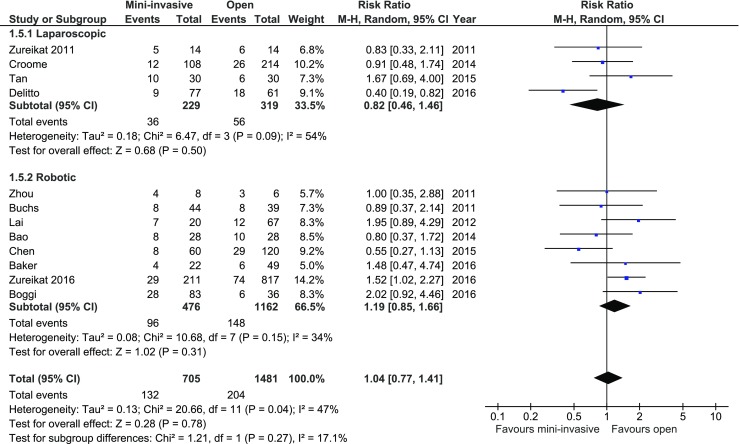
Pooled estimates of fistula cases comparing mini-invasive pancreatoduodenectomy versus open surgery. *CI* confidence interval, *df* degrees of freedom

All authors reported events of pancreatic fistula formation. International Study Group on Pancreatic Fistula (ISGPF) definition was used in 10 studies, while the remaining two described the definition of fistula in study methodology. Only one study, by Delitto et al., showed a significant difference in occurrence of fistula rate in favor of MIPD, whereas Zureikat et al. in their study from 2016 present data in favor of open approach. There were no significant variations among the studied groups: 132/705 (18.72%) in MIPD group vs. 204/1481 (13.77%) in control group, *RR* = 1.04, 95% CI 0.77–1.41, *p* for effect = 0.78, *p* for heterogeneity = 0.04, *I* ^2^ = 42% (Fig. 5).

**Fig. 6:**
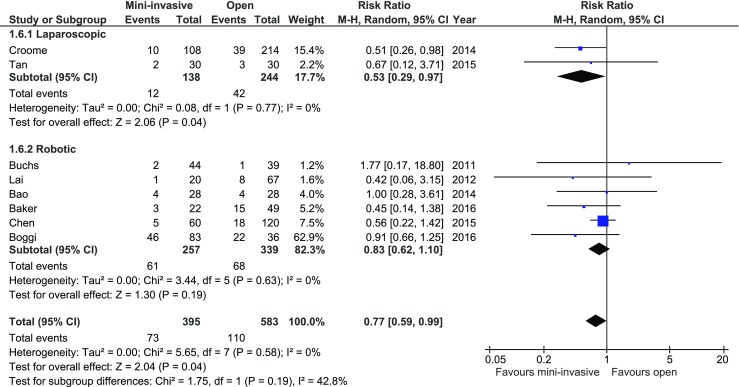
Pooled estimates of delayed gastric emptying cases comparing mini-invasive pancreatoduodenectomy versus open surgery. *CI* confidence interval, *df* degrees of freedom

Delayed gastric emptying was reported in eight studies. Only one, by Croome et al., showed a significant difference in rate of delayed gastric emptying in favor of MIPD. Subgroup analysis (Fig. 6) revealed a significant decrease in occurrence of delayed gastric emptying in the laparoscopic group (*p* = 0.04, 95% CI 0.29–0.97), whereas this was not present in the robotic subgroup (*p* = 0.19, 95% CI 0.62–1.1). In total, there was a significant variation in the rate of delayed gastric emptying with 73/395 (18.48%) in MIPD group and 110/583 (18.87%) in the open approach group: *RR* = 0.77, 95% CI 0.59–0.99, *p* for effect = 0.04, *p* for heterogeneity = 0.59, *I* ^2^ = 0%.

Postoperative hemorrhage was reported in six studies. None of the studies favored particular approach. There were no statistical significant differences in among analyzed groups: *RR* = 1.11, 95% CI 0.63–1.93, *p* for effect = 0.72, *p* for heterogeneity = 0.91, *I* ^2^ = 0%.

**Fig. 7:**
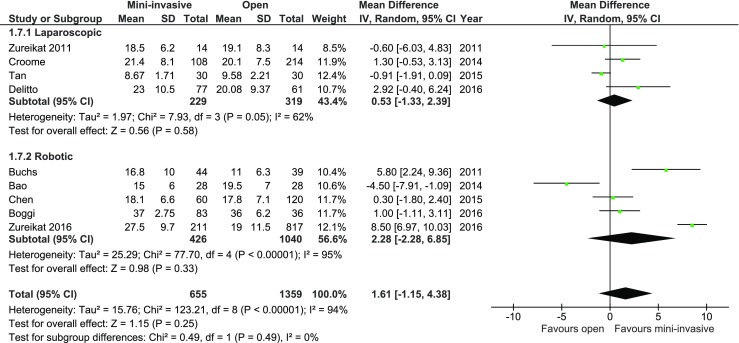
Pooled estimates of harvested lymph nodes comparing mini-invasive pancreatoduodenectomy versus open surgery. *CI* confidence interval, *df* degrees of freedom

The number of harvested lymph nodes was reported in nine studies. There were no significant variations among the analyzed groups, both in total and in subgroup analysis: MD = 1.61, 95% CI −1.15–4.38, *p* for effect = 0.25, *p* for heterogeneity <0.0001, *I* ^2^ = 94% (Fig. 7). Due to high heterogeneity, a sensitivity analysis was performed. Three studies by Buchs et al., Bao et al., and Zureikat et al. (2016) were identified as the cause for most heterogeneity. Analysis without these studies provided heterogeneity with *I* ^2^ = 45%; however, the result was still stastically insignificant, *p* = 0.44.

**Fig. 8:**
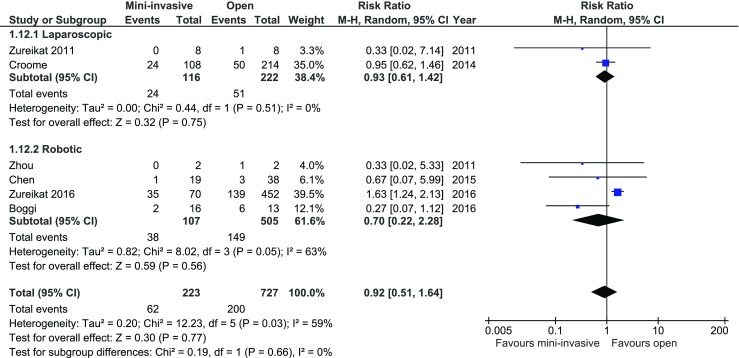
Pooled estimates of R1 resection comparing mini-invasive pancreatoduodenectomy versus open surgery. *CI* confidence interval, *df* degrees of freedom

R1 resection rate calculated separately for pancreatic malignancy was reported in 11/12; however, only in six extraction for pancreatic adenocarcinoma was possible. Of all studies, Zureikat et al. (2016) reported data in favor of open approach. Analysis of these six studies revealed no significant difference between the groups, 62/223 (27.80%) in MIPD vs. 200/727 (27.51%) in open approach group, *p* for effect *=* 0.77, *RR* = 0.92, 95% CI 0.51–1.64, *p* for heterogeneity = 0.03, *I* ^2^ = 59% (Fig. 8).

**Fig. 9:**
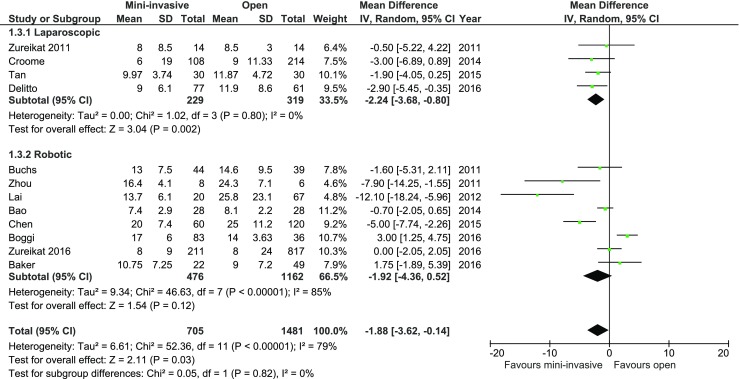
Pooled estimates of length of hospital stay comparing mini-invasive pancreatoduodenectomy versus open surgery. *CI* confidence interval, *df* degrees of freedom

Mean length of hospital (LOS) stay was reported in all papers, and in all of them, it included primary LOS (excluding potential readmissions). There was a significant reduction in LOS in the subgroup of laparoscopic surgery. Mean LOS for laparoscopic surgery was 8.2 days, while for the control group, it was 9.79 days (MD = −2.24, 95%CI −2.24–−0.80, *p* = 0.002). There were no statistical variations in LOS in the robotic group; however, authors Zhou, Lai, and Chen reported in their papers a significant reduction of LOS in the MIPD, whereas Boggi presented data favoring open approach. The analysis (Fig. 9) showed significant differences between studied groups in total: MD = −1.88, 95%CI −3.62–0.14, *p* for effect = 0.03, *p* for heterogeneity <0.0001, *I* ^2^ = 79%.

Thirty-day readmission rate was reported in three studies. The analysis revealed no stastically significant differences among analyzed groups, *RR* = 1.05, 95% CI 0.59–1.87, *p* for effect = 0.88, *p* for heterogeneity = 0.59, *I* ^2^ = 0%.

## Discussion

This systematic review, based on 12 comparative studies of minimally invasive pancreatoduodenectomy (four laparoscopic and eight robotic) enrolling 2186 patients, 705 in the MIPD group and 1481 in the open group, documents the feasibility and potential benefits of minimally invasive pancreatoduodenectomy. The subsequent meta-analysis of results showed that although MIPD takes longer, it is associated with a reduced intraoperative blood loss, lower incidence of delayed gastric emptying, and shorter LOS. Moreover, type of surgery had no influence on overall morbidity, pancreatic fistula rate, and number of harvested lymph nodes.

Laparoscopic PD was first described by Gagner and Pomp in 1994, whereas first reports on robotic approach appeared 6 years later [1, 31]. Despite high popularity of minimally invasive surgery in other surgical disciplines and all its undeniable benefits, MIPD has still not become the gold standard in the treatment of periampullary tumors [32, 33]. While performing this systematic review, we did not find any randomized controlled trials on this topic; thus, the quality of included studies is limited. To our knowledge, there are several previously published systematic reviews comparing MIPD with open approach [34–36]. However, in most of them, there is a methodological bias due to the fact that they include in their analysis studies which apart from pancreatoduodenectomy report pancreatectomies altogether. They do not report parameters such as operative time, blood loss, pancreatic fistula, overall morbidity, and LOS separately for pancreatoduodenectomy and pancreatectomy, which makes previous meta-analyses including these studies prone to be biased. Therefore, in our review, we included only studies reporting clear data on MIPD, thus, limiting bias associated with uncertain data.

MIPD is considered one of the most difficult and time-consuming abdominal procedures, since it involves precise perivascular dissection and demanding biliary and pancreatic anastomoses. For these reasons, in some centers, the dissection is the only part performed via laparoscopy, whereas anastomoses are constructed via minilaparotomy, which is obviously less complicated and time-consuming than with laparoscopic instruments. Therefore, to fully compare MIPD and open approach, we have decided to exclude all studies which described hand-assisted methods. Studies in which the method description was unclear or it was stated that anastomoses were performed extra- or intracorporeal (up to surgeon’s decision) were excluded.

Mean tumor size in the MIPD group was significantly lower than in open group. This is probably due to case selection for MIPD and, as long as there are no RCTs on this topic, tumor size may create potential methodological bias. Other tumor characteristics, such as malignant/benign character or stage of cancer did not vary.

We have observed that operative time was longer in MIPD group (464 vs. 388 min), which confirms the difficulty of the procedure. Previous studies have estimated that at least 50 cases for laparoscopic and 33 (up to 80 in one report) for robotic access are needed to achieve proficiency in performing them [24, 37–39]. However, in all of them, operative time was taken into consideration to establish the completion of the learning curve. After all, achieving proficiency in such a difficult procedure cannot be limited only to reduction of operative time. It should also include other parameters such as pancreatic fistula rate, intraoperative blood loss, or other postoperative complications. Unfortunately, in most of selected studies, the experience of the surgical team was unclear, and it was not stated whether the surgical team has completed the learning curve, and for this reason, analysis of outcomes may be biased. In addition, there is a tendency that even beyond the completion of defined learning curves, a selection bias may be present. Therefore, well-matched trials or randomization is needed to eliminate this element. On the other hand, reduced blood loss seems typical for minimally invasive procedures, since it is desired for appropriate visualization of the operating field. It is not surprising that most of the studies reporting blood loss showed its reduction in MIPD groups.

Perhaps the most important aspect from a surgeon’s perspective is the comparison of complication rates, LOS, and oncological outcomes. Our review did not find any differences in overall morbidity between MIPD and OPD. Although we have found that delayed gastric emptying rate was statistically lower in MIPD group, the difference was clinically irrelevant (18.48 vs. 18.87%). Pancreatic fistula rates remained similar, irrespective of surgical approach. Even though available evidence of included studies suggests that MIPD does not significantly improve outcomes in terms of morbidity, it may guarantee the possibility of noninferiority. This is also confirmed by a similar number of harvested lymph nodes in both groups. Additionally, we have observed that R1 rate was similar in both groups. However, it has to be emphasized that there were differences in baseline tumor sizes between groups, which with no doubt biases this result. Indeed, it is well-established that tumor size is an important feature of pancreatic head cancer and correlates strongly with incomplete resection rate [40, 41]. Besides, there was no information on the pathology protocol, which could vary among studies, leading to biased results [42–44]. Moreover, an overall reduction in LOS was seen but with relatively high heterogeneity. This may be explained by other factors that are related to LOS (perioperative care protocols and different discharge criteria).

Our review has some important limitations which are related to available studies. It does not consist of any randomized study, which limits the level of evidence. In addition, it contains results of several specialized, high-volume centers and probably cannot be easily transferred to departments with lower annual case volume. Moreover, high statistical heterogeneity was found among included studies. For some parameters, it reached 90% which limits the quality of results and may even question performing such meta-analysis. However, we have decided to show that there is still little evidence behind potential benefits of MIPD. The analyzed studies may be prone to selection bias as a difference in the tumor size between groups has been documented. All these aspects are difficult to avoid in cohort studies, and therefore well-designed randomized trials are necessary to fully answer the basic question: what are the potential benefits associated with the introduction of minimally invasive surgery to surgical treatment of periampullary tumors. Although we included only MIPD groups, we did not analyze the surgical technique (artery first approach vs. traditional, types of pancreatic and biliary anastomoses, perioperative care used). This obviously may have a significant impact on final results as previously suggested [45–47].

## Conclusions

The findings of this systematic review and subsequent meta-analysis shows that minimally invasive pancreatic head surgery is not ready for general application and should be performed in specialized high-volume pancreatic centers with extensive expertise in minimally invasive surgery. Our review suggests that although MIPD takes longer, it may be associated with reduced blood loss, shortened LOS, and comparable rate of perioperative complications. However, it is based on non-randomized studies only of high-volume specialized centers. The differences in baseline characteristics of patients may possibly lead to a high risk of selection bias. In our opinion, the existing evidence for the use of laparoscopic surgery in pancreatic head malignancy is very limited and should be interpreted with caution. This supports the concept that further, better quality studies are needed to provide higher level of evidence on the benefits of minimally invasive approach in pancreatic head surgery.

## Electronic supplementary material

Supplementary file 1 (GIF 127 kb)

High resolution image (TIFF 28 kb)

## References

[1] Gagner M, Pomp A (1994). Laparoscopic pylorus-preserving pancreatoduodenectomy. Surg Endosc.

[2] Lassen K, Coolsen MME, Slim K (2012). Guidelines for perioperative care for pancreaticoduodenectomy: enhanced recovery after surgery (ERAS®) society recommendations. World J Surg.

[3] Correa-Gallego C, Dinkelspiel HE, Sulimanoff I (2014). Minimally-invasive vs open pancreaticoduodenectomy: systematic review and meta-analysis. J Am Coll Surg.

[4] Song KB, Kim SC, Hwang DW (2015). Matched case-control analysis comparing laparoscopic and open pylorus-preserving pancreaticoduodenectomy in patients with periampullary tumors. Ann Surg.

[5] Wellner UF, Küsters S, Sick O (2014). Hybrid laparoscopic versus open pylorus-preserving pancreatoduodenectomy: retrospective matched case comparison in 80 patients. Langenbeck’s Arch Surg.

[6] Mendoza AS, Han H-S, Yoon Y-S (2015). Laparoscopy-assisted pancreaticoduodenectomy as minimally invasive surgery for periampullary tumors: a comparison of short-term clinical outcomes of laparoscopy-assisted pancreaticoduodenectomy and open pancreaticoduodenectomy. J Hepatobiliary Pancreat Sci.

[7] Wang Y, Bergman S, Piedimonte S, Vanounou T (2014). Bridging the gap between open and minimally invasive pancreaticoduodenectomy: the hybrid approach. Can J Surg.

[8] Walsh RM, Chalikonda S (2016). How I do it: hybrid laparoscopic and robotic pancreaticoduodenectomy. J Gastrointest Surg.

[9] Inoue Y, Saiura A, Sato T (2016). Laparoscopic pancreatoduodenectomy combined with a novel self-assessment system and feedback discussion: a phase 1 surgical trial following the IDEAL guidelines. Langenbeck’s Arch Surg.

[10] Arezzo A, Passera R, Salvai A (2015). Laparoscopy for rectal cancer is oncologically adequate: a systematic review and meta-analysis of the literature. Surg Endosc.

[11] Quan Y, Huang A, Ye M (2016). Comparison of laparoscopic versus open gastrectomy for advanced gastric cancer: an updated meta-analysis. Gastric Cancer.

[12] Hozo SP, Djulbegovic B, Hozo I (2005). Estimating the mean and variance from the median, range, and the size of a sample. BMC Med Res Methodol.

[13] Stroup DF, Berlin JA, Morton SC et al (2000) Meta-analysis of observational studies in epidemiology: a proposal for reporting. Meta-analysis of observational studies in epidemiology (MOOSE) group. JAMA 283(15):2008–2012 10.1001/jama.283.15.2008 10789670

[14] Moher D, Liberati A, Tetzlaff J (2009). Preferred reporting items for systematic reviews and meta-analyses: the PRISMA statement. PLoS Med.

[15] Asbun HJ, Stauffer JA (2012). Laparoscopic vs open pancreaticoduodenectomy: overall outcomes and severity of complications using the Accordion Severity Grading System. J Am Coll Surg.

[16] Mesleh MG, Stauffer JA, Bowers SP, Asbun HJ (2013). Cost analysis of open and laparoscopic pancreaticoduodenectomy: a single institution comparison. Surg Endosc.

[17] Stauffer JA, Coppola A, Villacreses D (2016). Laparoscopic versus open pancreaticoduodenectomy for pancreatic adenocarcinoma: long-term results at a single institution. Surg Endosc.

[18] Gumbs AA, Grès P, Madureira FA, Gayet B (2008). Laparoscopic vs. open resection of noninvasive intraductal pancreatic mucinous neoplasms. J Gastrointest Surg.

[19] Baker EH, Ross SW, Seshadri R (2016). Robotic pancreaticoduodenectomy: comparison of complications and cost to the open approach. Int J Med Rob Comput Assisted Surg.

[20] Bao PQ, Mazirka PO, Watkins KT (2014). Retrospective comparison of robot-assisted minimally invasive versus open pancreaticoduodenectomy for periampullary neoplasms. J Gastrointest Surg.

[21] Boggi U, Napoli N, Costa F (2016). Robotic-assisted pancreatic resections. World J Surg.

[22] Buchs NC, Addeo P, Bianco FM (2011). Robotic versus open pancreaticoduodenectomy: a comparative study at a single institution. World J Surg.

[23] Chen S, Chen J-Z, Zhan Q (2015). Robot-assisted laparoscopic versus open pancreaticoduodenectomy: a prospective, matched, mid-term follow-up study. Surg Endosc.

[24] Lai ECH, George PCY, Tang CN (2012). Robot-assisted laparoscopic pancreaticoduodenectomy versus open pancreaticoduodenectomy—a comparative study. Int J Surg.

[25] Zhou NX, Chen JZ, Liu Q (2011). Outcomes of pancreatoduodenectomy with robotic surgery versus open surgery. Int J Med Rob Comput Assisted Surg.

[26] Zureikat AH, Postlewait LM, Liu Y (2016). A multi-institutional comparison of perioperative outcomes of robotic and open pancreaticoduodenectomy. Ann Surg.

[27] Croome KP, Farnell MB, Que FG (2014). Total laparoscopic pancreaticoduodenectomy for pancreatic ductal adenocarcinoma. Ann Surg.

[28] Delitto D, Luckhurst CM, Black BS (2016). Oncologic and perioperative outcomes following selective application of laparoscopic pancreaticoduodenectomy for periampullary malignancies. J Gastrointest Surg.

[29] Tan C-L, Zhang H, Peng B, Li K-Z (2015). Outcome and costs of laparoscopic pancreaticoduodenectomy during the initial learning curve vs laparotomy. World J Gastroenterol.

[30] Zureikat AH, Breaux JA, Steel JL, Hughes SJ (2011). Can laparoscopic pancreaticoduodenectomy be safely implemented?. J Gastrointest Surg.

[31] Giulianotti PC, Sbrana F, Bianco FM (2010). Robot-assisted laparoscopic pancreatic surgery: single-surgeon experience. Surg Endosc.

[32] de Rooij T, Klompmaker S, Abu Hilal M (2016). Laparoscopic pancreatic surgery for benign and malignant disease. Nat Rev Gastroenterol Hepatol.

[33] Coppola A, Stauffer JA, Asbun HJ (2016). Laparoscopic pancreatoduodenectomy: current status and future directions. Updat Surg.

[34] Zhang H, Wu X, Zhu F (2016). Systematic review and meta-analysis of minimally invasive versus open approach for pancreaticoduodenectomy. Surg Endosc.

[35] Wang M, Cai H, Meng L (2016). Minimally invasive pancreaticoduodenectomy: a comprehensive review. Int J Surg.

[36] Qin H, Qiu J, Zhao Y (2014). Does minimally-invasive pancreaticoduodenectomy have advantages over its open method? A meta-analysis of retrospective studies. PLoS One.

[37] Speicher PJ, Nussbaum DP, White RR (2014). Defining the learning curve for team-based laparoscopic pancreaticoduodenectomy. Ann Surg Oncol.

[38] Boone BA, Zenati M, Hogg ME (2015). Assessment of quality outcomes for robotic pancreaticoduodenectomy: identification of the learning curve. JAMA Surg.

[39] Napoli N, Kauffmann EF, Palmeri M (2016). The learning curve in robotic pancreaticoduodenectomy. Dig Surg.

[40] Petermann D, Demartines N, Schäfer M (2013). Is tumour size an underestimated feature in the current TNM system for malignancies of the pancreatic head?. HPB (Oxford).

[41] Marchegiani G, Andrianello S, Malleo G (2016). Does size matter in pancreatic cancer?: reappraisal of tumour dimension as a predictor of outcome beyond the TNM. Ann Surg.

[42] Esposito I, Kleeff J, Bergmann F (2008). Most pancreatic cancer resections are R1 resections. Ann Surg Oncol.

[43] Verbeke C, Löhr M, Karlsson JS, Del Chiaro M (2015). Pathology reporting of pancreatic cancer following neoadjuvant therapy: challenges and uncertainties. Cancer Treat Rev.

[44] Verbeke CS, Gladhaug IP (2012). Resection margin involvement and tumour origin in pancreatic head cancer. Br J Surg.

[45] Hackert T, Werner J, Weitz J (2010). Uncinate process first—a novel approach for pancreatic head resection. Langenbeck’s Arch Surg.

[46] Witzigmann H, Diener MK, Kienkötter S (2016). No need for routine drainage after pancreatic head resection: the dual-center, randomized, controlled PANDRA trial (ISRCTN04937707). Ann Surg.

[47] Sanjay P, Takaori K, Govil S (2012). “Artery-first” approaches to pancreatoduodenectomy. Br J Surg.

