# Supplementary figures and images for: Minimally invasive versus open pancreatoduodenectomy—systematic review and meta-analysis
Source: Langenbecks Arch Surg. 2017 May 9;402(5):841–51. doi: 10.1007/s00423-017-1583-8 (PMC5506213; doi:10.1007/s00423-017-1583-8)

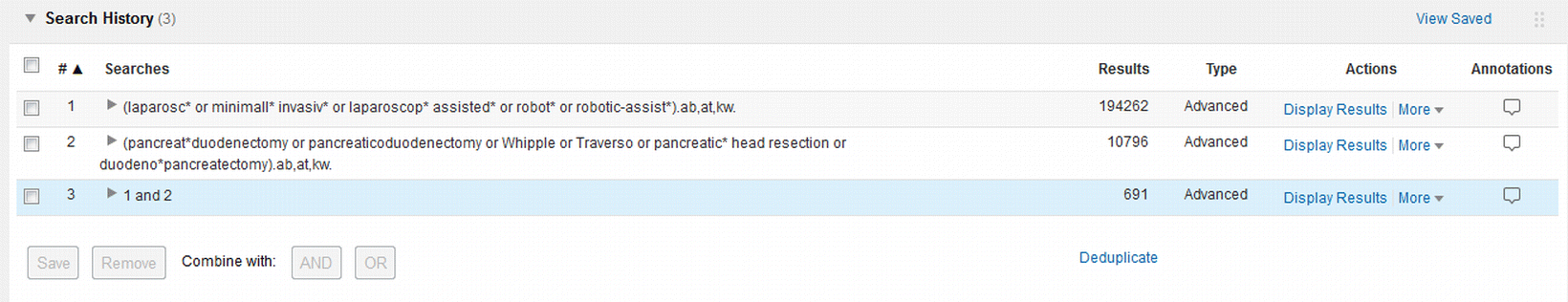

Supplement: Supplementary file 1 — (GIF 127 kb) [file 423_2017_1583_Fig10_ESM.gif]

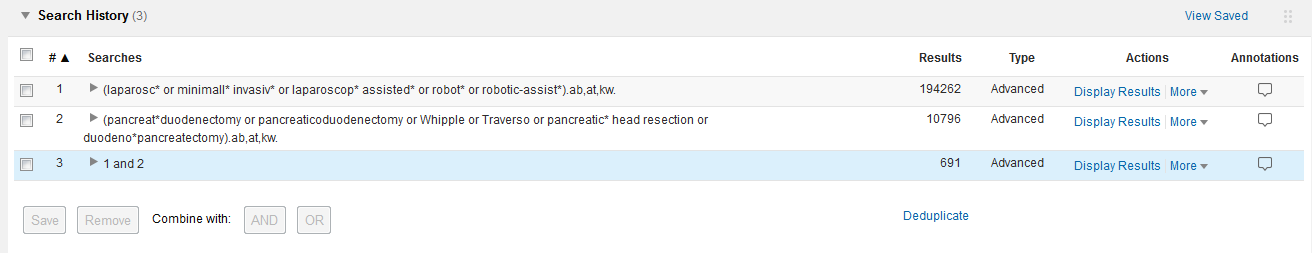

Supplement: Supplementary file 2 — High resolution image (TIFF 28 kb) [file 423_2017_1583_MOESM1_ESM.tif]
